# Does Position Affect Reduction? Comparison of the Effects of Three Different Positions on Reduction in Intertrochanteric Femur Fracture Nailing

**DOI:** 10.3390/medicina61061005

**Published:** 2025-05-28

**Authors:** Nezir Okumuş, Ahmet Nadir Aydemir

**Affiliations:** 1 Denipol Hospital, Denizli 20010, Türkiye; nezir.okumus@gmail.com; 2 Faculty Depth of Orthopaedics, Pamukkale University Medicine, Denizli 20160, Türkiye

**Keywords:** proximal femoral nail, traction table, lateral decubitus, supine position

## Abstract

*Background and Objectives*: Our study aimed to retrospectively examine the routine radiographs on the first postoperative day of osteosynthesis applications performed in the supine position with the help of a traction table, in the lateral decubitus position, and in the supine position in patients with intertrochanteric fractures of the femur who had a proximal femoral nail applied. It also aimed to compare them in terms of radiology. This study investigated the effects of three different patient positions on fracture reduction, a topic rarely encountered in the literature. *Materials and Methods*: Patients who underwent proximal femoral nailing in three different positions—the supine, traction table, and lateral decubitus positions—due to femoral intertrochanteric fractures in two different centers were analyzed. A total of 157 patients with complete early radiographs were included in this study to evaluate the quality of postoperative reduction and fixation. *Results*: There was a significant difference between the traction table-assisted supine position group (mean: 25.31 mm) and both the lateral decubitus position (mean: 31.91 mm) and supine position (mean: 31.79 mm) groups in terms of the TAD (*p* = 0.000). Regarding the collodiaphyseal angle, the traction table-assisted supine position (mean: 130.720°) and lateral decubitus position (mean: 130.290°) groups showed significantly higher values than the supine position group (mean: 124.190°) (*p* = 0.000). The average lengths of the lag and compression screws were lower in the lateral decubitus position group compared with the other groups (*p* = 0.000). Patients in the supine position group had smaller nail diameters and lengths (*p* = 0.000). When examining the Cleveland–Bosworth lag screw placements, the most frequent position was center–center, including 22 patients (31%) in the traction table-assisted supine position group, 15 patients (30.6%) in the lateral decubitus position group, and 9 patients (24.3%) in the supine position group, though the difference was not statistically significant (*p* = 0.203). Among the reduction criteria we investigated, the TAD on the traction table was statistically significantly closer to the targeted measurement, with an average of 25.31 mm, compared with the other two positions (*p* = 0.000). The collodiaphyseal angle was significantly within the target range in the traction table-assisted supine group, averaging 130.720°, compared with the supine position (*p* = 0.000). In the traction table group, according to the modified Baumgaertner classification, 59.2% achieved a good reduction; according to the Ikuta classification, subtype N accounted for 69.4%; and according to the Cleveland–Bosworth classification, a center–center placement was present in 31% of patients. *Conclusions*: All three types of operation can be preferred according to the habits of the surgeon operating and the variables during the operation (the fracture type, history of orthopedic surgery, and the material components of the application phase). Accompanied by these data, we recommend the traction table operation as a priority and the lateral decubitus position operation as a second preference in compliance with the technical requirements.

## 1. Introduction

Intertrochanteric femur fractures are among the most common hip fractures, particularly in elderly patients with osteoporotic bones, usually due to low-energy trauma such as simple falls. The incidence of intertrochanteric femur fractures has significantly increased in recent years, and this trend is expected to continue soon due to the increasing geriatric population and the rising incidence of osteoporosis [1].

An alternative method for the fixation of extracapsular hip fractures is an intramedullary nail (PFN). A theoretical advantage of intramedullary fixation is the biomechanical stability achieved by reducing the distance between the hip joint and the implant. However, a significant disadvantage is the stress that occurs distal to the distal locking screws [2]. Cephalomedullary nails are the preferred implants for treating most extracapsular proximal femur fractures [3].

Traction tables are the most frequently preferred method for the surgical treatment of intertrochanteric fractures [3]. The primary advantage of this technique is the ability to achieve indirect reduction via continuous and consistent traction throughout the surgical procedure. Nevertheless, traction tables may lead to complications, such as limb length discrepancy, malrotation, nerve neuropraxia resulting from prolonged traction, dermal necrosis due to pressure from the perineal post, and compartment syndrome in the contralateral healthy limb [4]. To avoid these potential complications associated with traction tables, patients may alternatively undergo surgery in the lateral decubitus position, which was first introduced by Davis in 1969 for proximal femur fractures [5]. An additional advantage of this position is improved access to the greater trochanter, particularly beneficial in obese patients. However, this approach may pose disadvantages, such as intolerance in patients with unstable spinal fractures or thoracic trauma and increased difficulty in managing anesthetic emergencies during the operation [6]. As a third surgical option, patients can undergo the procedure in the supine position without using a traction table. This approach avoids specific complications related to traction tables and offers benefits such as shorter operative times. However, challenges include difficulty obtaining adequate lateral fluoroscopic views of the proximal femur, maintaining consistent traction, and the necessity for additional surgical assistance [6,7].

Although the effects of these three approaches on reduction criteria for proximal femur nail application have been individually examined in previous studies, no studies that simultaneously conducted radiological comparisons of all three application methods for proximal femur nail procedures have been found.

This study aimed to retrospectively examine and compare the routine radiographs of patients with intertrochanteric femur fractures treated with proximal femur nails operated on in the traction table-assisted supine position, lateral decubitus position, and supine position without traction, taken on the first postoperative day, from a radiological perspective. Our study’s main questions were as follows: Does patient positioning affect the fracture reduction? If so, which position demonstrates superior reduction success compared with the others?

## 2. Materials and Methods

This study was carried out retrospectively by examining patient data from two different centers following approval by the Pamukkale University Ethics Committee (approval number: 289685; date: 15 March 2022). The patients included in this study underwent surgery for femoral intertrochanteric fractures at two different hospitals: one university hospital’s Orthopedics and Traumatology Clinic, performing operations in the supine position using a traction table, and one state hospital’s Orthopedics and Traumatology Clinic, performing operations using the Smith & Nephew Trigen Intertan system in both the lateral decubitus and supine positions. The surgeries were performed by orthopedic surgeons with at least 5 years of surgical experience. Patients diagnosed with intertrochanteric femur fractures and treated surgically using the Smith & Nephew Trigen InterTAN system over four years at two different centers were included in this study. Patients who underwent an open reduction or whose early postoperative radiographs were unavailable were excluded.

Operation with Supine Position Assisted by Traction Table

**Figure 1:**
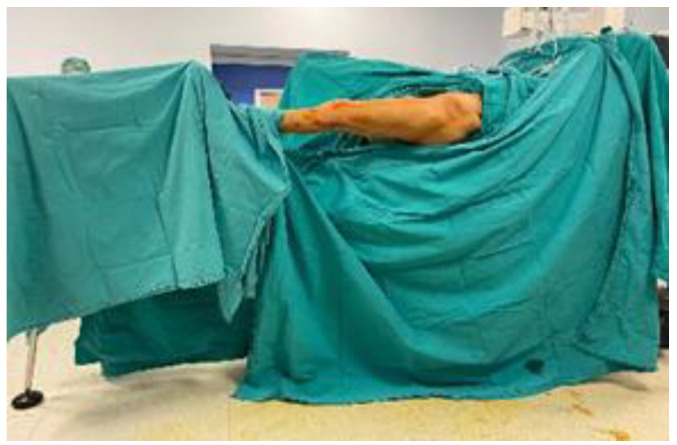
Operation in supine position assisted by traction table.

Patients were positioned supine on an operating table equipped with a traction apparatus, with the unaffected leg in the lithotomy position and the affected leg secured in the traction device, stabilized under spinal and/or general anesthesia. Reduction was achieved by applying longitudinal traction and internal rotation via the traction device. Reduction quality was evaluated using fluoroscopy images from both the anteroposterior and lateral views (Figure 1).

Operation in Lateral Decubitus Position

**Figure 2:**
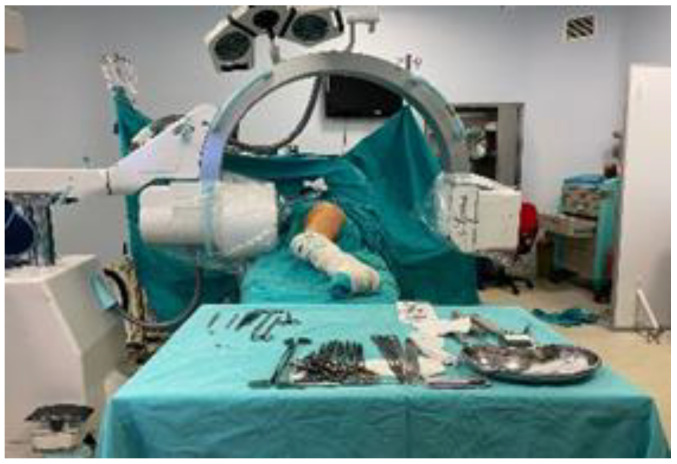
Operation in lateral decubitus position.

Patients were positioned in the lateral decubitus position on a standard operating table, stabilized with lateral support bars, under spinal and/or general anesthesia. Reduction quality was evaluated using fluoroscopic anteroposterior and lateral images. The lateral view was taken with the hip in approximately 90 degrees of flexion and 30–40 degrees of abduction (Figure 2).

Operation in Supine Position

**Figure 3:**
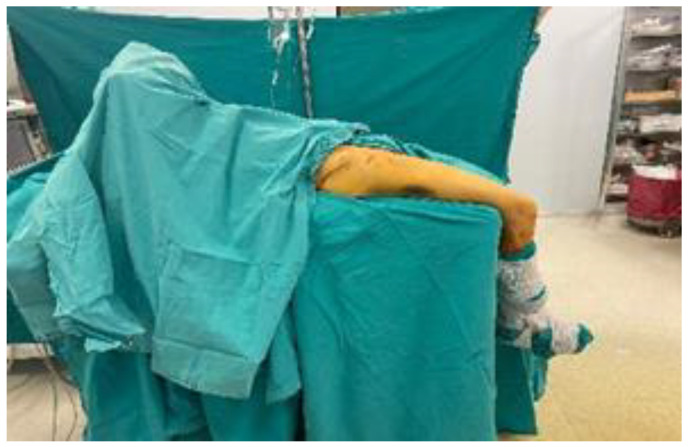
Operation in supine position.

Patients were positioned supine on a standard operating table, with both legs hanging freely at the knee joints, stabilized under spinal and/or general anesthesia. Reduction was achieved by gravitational muscle forces acting on the traumatized extremity. Reduction quality was evaluated using fluoroscopic anteroposterior images only. Lateral imaging could not be performed due to superimposition (Figure 3).

The study groups were defined as follows: Group I included patients operated on in the supine position assisted by a traction table; Group II consisted of patients operated on in the lateral decubitus position; and Group III comprised patients operated on in the supine position. The preoperative and postoperative day 1 routine radiographs of all patients across the three groups were independently evaluated by two surgeons. The evaluators assessed the radiographs twice, one week apart, without knowing the center or the patient’s position during surgery.

The following measurements were assessed in the radiological evaluations of patients with proximal femur fractures treated with the Smith & Nephew Trigen InterTAN system: the tip–apex distance (TAD), using standardized lag screw lengths of Dtrue (AP) 15.25 mm and Dtrue (lateral) 10.8 mm for magnification correction in the calculation [8]; the collodiaphyseal angle (CAD) [9]; the Cleveland and Bosworth classification [10]; the Ikuta classification [11]; and the modified Baumgaertner classification [12]. These radiological parameters were selected both to assess the quality of the fracture reduction and to predict the risk of cut-out or mechanical failure during follow-up, even in cases where the reduction appeared satisfactory.

### Statistical Analysis

The data were analyzed using SPSS 25.0 (IBM SPSS Statistics 25 software, IBM Corp.: Armonk, NY, USA). Continuous variables were presented as means ± standard deviations, and categorical variables as numbers and percentages. To compare differences between independent groups, a one-way ANOVA was used if parametric assumptions were met; otherwise, the Kruskal–Wallis test was applied. For paired-group comparisons, the paired-samples *t*-test was employed if parametric assumptions were met; otherwise, the Wilcoxon test was utilized. A *p*-value of <0.05 was considered statistically significant.

## 3. Results

Of the patients included in this study, 97 (61.8%) were female and 60 (38.2%) were male. When the distribution of patients according to the type of operation performed was examined, 71 patients (45.2%) were treated on the traction table, 49 (31.2%) in the lateral decubitus position, and 37 (23.6%) in the supine position. The average age of the patients was 72.32. When the relationships between the types of surgical procedures and the AO, modified Baumgaertner, and Ikuta classifications were examined, no statistically significant differences were found. The associations between the groups and the classifications are presented in Table 1.

**Figure 4:**
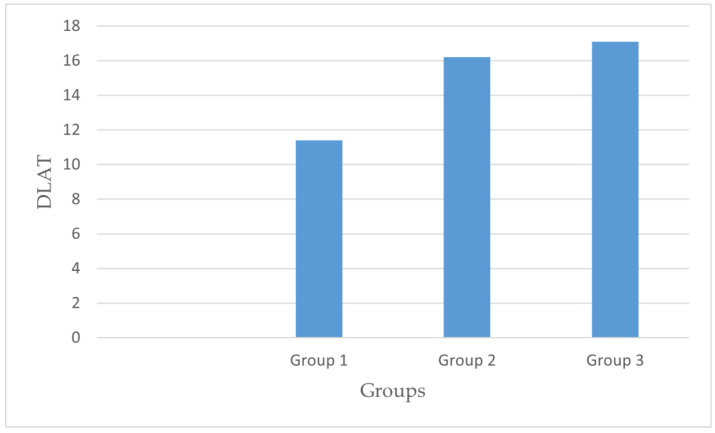
DLAT comparison across groups.

**Figure 5:**
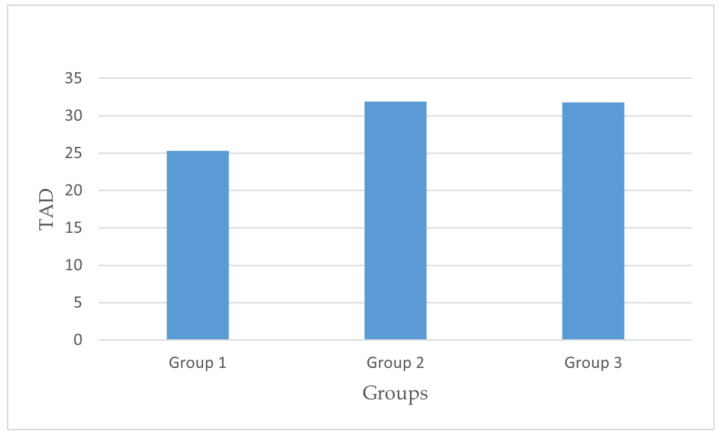
TAD comparison across groups.

When evaluating the surgical technique in terms of the anterior–posterior distance (DAP), the mean values were 13.88 ± 4.48 mm in Group I, 15.72 ± 5.68 mm in Group II, and 15.20 ± 6.72 mm in Group III, with no statistically significant difference found (*p* = 0.156). For the lateral distance (DLAT) measurements based on the surgical technique, the mean values were 11.43 ± 4.10 mm in Group I, 16.21 ± 7.08 mm in Group II, and 17.11 ± 6.30 mm in Group III, with a statistically significant difference observed (*p* = 0.000). Regarding the TAD, the mean values were 25.31 ± 6.62 mm in Group I, 31.91 ± 11.32 mm in Group II, and 31.79 ± 11.14 mm in Group III, indicating a statistically significant difference (*p* = 0.000). The TAD and DLAT measurements are presented in Figure 4 and Figure 5. When examining the surgical technique and collodiaphyseal angle, the mean values were 130.72 ± 6.38° in Group I, 130.29 ± 8.04° in Group II, and 124.19 ± 6.41° in Group III, showing a statistically significant difference (*p* = 0.000). In Group I, the lag screw was positioned closer to the apex in both the anteroposterior and lateral planes compared with the other groups. The findings are presented in Table 2.

A statistically significant difference was found when comparing the lag screw length and compression screw length based on the surgical technique (*p* < 0.000). In Group I, both the lag screw and the compression screw were placed longer compared with the other groups. The findings are presented in Table 3.

When the surgical technique was evaluated in relation to the Cleveland–Bosworth positioning, 22 patients (31%) in Group I, 15 (30.6%) in Group II, and 9 (24.3%) in Group III had a center–center placement. No statistically significant difference was found between the groups (*p* = 0.203).

## 4. Discussion

Our study aimed to compare the reduction parameters in PFN surgeries performed using three different techniques. Based on our results, the use of a traction table is our preferred choice, provided that appropriate resources and conditions are available.

The quality of reduction was assessed in three categories based on a modification of the classification originally developed by Baumgaertner et al. [13]. The first criterion utilized was an AP neck–shaft angle between 120° and 135°, combined with a lateral angulation of less than 20°. The second criterion was the presence of fragment displacement of less than 4 mm in the AP and lateral radiographic views. If both criteria were satisfied, the reduction was categorized as ‘good’; if only one criterion was met, it was categorized as ‘acceptable’; and if neither criterion was fulfilled, it was classified as ‘poor’. Patients with poor reductions have been reported to have a more than threefold higher rate of cut-out compared with those with good reductions. In a study from India evaluating PFN surgeries performed in the lateral decubitus position, using the modified Baumgaertner classification, it was reported that 90% of patients had good reductions, while 10% had acceptable reductions [14]. Another study conducted in the same region reported that 92% of patients operated in the supine position had good reductions, with no cases classified as poor [15]. A comparative study from 2016 evaluating traction table and lateral decubitus positions reported good reductions in 69.4% and poor reductions in 5.5% of patients operated on using the traction table. Meanwhile, in the lateral decubitus group, 40.54% had good reductions, and 10.81% had poor reductions [16]. In our study, poor reductions, according to the modified Baumgaertner classification, were most frequently observed in Group III (10.8%), followed by Group II (4.1%) and Group I (2.8%). Good reductions were observed in 63.4% of Group I, 59.2% of Group II, and 62.2% of Group III.

Although previous studies have established that the quality of fracture reduction can influence postoperative outcomes, these primarily focused on evaluations using AP radiographs, and there remains no consensus regarding the assessment of the fracture reduction quality using lateral radiographs [17]. According to the Ikuta classification, posterior displacement of the head–neck junction (subtype P) is associated with an increased likelihood of reduction loss in the postoperative period [18]. Tsukada et al. demonstrated, using statistical analysis, that excessive sliding after surgical treatment of pertrochanteric fractures is related to postoperative subtype P [19]. Tagikawa et al. reported that the rate of Ikuta subtype N was 83% among patients operated on using a traction table, while the rate of Ikuta subtype P was 3.3% in patients classified as Evans–Jensen types 1-2, increasing to 20.5% in Evans–Jensen types 3-4 fractures [20]. Turgut et al. reported Ikuta subtype N in 85%, subtype P in 7.2%, and subtype A in 7.7% of patients operated in the lateral decubitus position, with a cut-out incidence of 4% [3]. In our study, subtype N placement averaged 64.8% in Group I, 69.4% in Group II, and 64.9% in Group III. Patients with subtype P were more frequent in Group III (21.6%) compared with the other two groups.

Cut-out or implant failure in the lag screw may result in long-term pain in the affected limb. As reported by Takigami et al., achieving early full ambulation in the presence of an insufficient lag screw insertion depth may lead to lag screw cut-out, reporting an incidence of 2% in their study [21]. Additionally, when a single lag screw is used, the screw may act as a source of rotational instability within the bone, potentially causing loosening at the bone–screw interface and subsequent cut-out [22]. Clinical studies related to biomechanics have demonstrated good anti-rotation performance in procedures employing double-blade lag screws. In a study where the nail lengths ranged from a minimum of 200 mm to a maximum of 240 mm, the lag screw lengths were reported to range between 75 mm and 120 mm [23]. In our study, the lag screw lengths in Group I (mean: 97.21 mm) were statistically significantly longer compared with the other two groups. We attribute this to the traction table’s ability to restore the hip offset closer to normal after reduction.

TAD has become a widely preferred criterion for determining the position of the hip screw [12]. It is calculated by adding the magnification-adjusted distance measured from the tip of the implant to the femoral head’s medial border along an imaginary line through the center of the femoral head in the anteroposterior radiograph to the distance obtained using the same method in the lateral radiograph. However, the optimal TAD value for PFN procedures performed with double-blade screws is still debated [24]. In a study group composed of patients operated on in the lateral decubitus position, the authors reported TAD values of less than 20 mm in 79% of cases, but they did not specify the TAD values in cut-out cases [25]. Penzkofer et al. reported an average TAD of 28 mm and three cases of cut-out among 66 patients treated with PFNA in the lateral decubitus position for intertrochanteric and subtrochanteric fractures [26]. Mereddy et al. reported a cut-out rate of 3.6% in a series of 62 cases treated with PFN in the lateral decubitus position, 70% of whom were female, predominantly consisting of 31 A2 and A3 fractures. They reported a center–center lag screw position in 52 cases, with an average TAD of 12 mm (range: 4–34 mm) [25]. Another study with a mean TAD value of 17.97 mm (range: 11–33 mm) found failure in 2 out of 93 cases (2.15%) with a TAD of <25 mm and in 1 out of 7 cases (14.28%) with a TAD of >25 mm [8]. Geller et al. reported a 10% cut-out rate among patients with a TAD above 25 mm, representing 44% of all their cases [27]. Duramaz et al., in their 2019 study comparing patients operated on with Intertan, PFNA-II, and Profin on a traction table, reported an average TAD of 26.7 mm in the Intertan group [28]. In our study, Group I demonstrated a favorable mean TAD value (25.31 mm), significantly lower compared with Groups II and III, which had mean TAD values of 31.91 mm and 31.79 mm, respectively. However, it remains impossible to determine which individual measurement (AP or LAT) contributed more to the TAD, defined as TAD = DAP + DLAT. Our literature review indicated that separate analyses of DAP and DLAT values were not conducted when calculating the TAD from radiographic images. Therefore, our study included separate statistical analyses of both DAP and DLAT values. The mean DLAT value in Group I (11.43 mm) was significantly lower compared with Group II (16.21 mm) and Group III (17.11 mm), a difference that influenced the overall TAD values. We attribute this discrepancy mainly to the difficulty in obtaining accurate intraoperative lateral fluoroscopic images in patients operated on in the lateral decubitus and supine positions. Future studies should investigate the relationship between DAP-DLAT values and outcomes such as cut-out rates, implant failure, postoperative pain, ambulatory success, and other clinical scores.

The risk of varus collapse in the late postoperative period can be anticipated using the collodiaphyseal angle measurement [29]. The collodiaphyseal angle is defined as the angle formed between the anatomical axis of the femoral neck and the anatomical axis of the femoral diaphysis. In a series of 225 cases with intertrochanteric fractures treated with PFN, the authors reported an average collodiaphyseal angle of 128°, with varus collapse observed in 4.9% of patients [30]. Another study, in which patients were operated on in the supine position, reported varus collapse in 12% of cases, despite 88% of the patients having a neck–shaft angle of greater than 120 degrees [31]. Following PFN surgery performed with traction table assistance in the supine position, patients who experienced cut-out had an average collodiaphyseal angle of 137° [32]. Similarly, in our study, the collodiaphyseal angle was comparable between Groups I (mean: 130.72°) and II (mean: 130.29°), whereas Group III exhibited a lower average angle of 124.19°.

The Cleveland–Bosworth classification is defined by dividing the femoral head, represented as a sphere in lateral radiographs, into nine equal quadrants and determining the location of the lag screw tip within these quadrants [14]. Numerous studies have demonstrated that the optimal positioning of the lag screw within the femoral head is a critical factor in preventing mechanical failure of osteosynthesis [33]. Sharma et al. reported that 17 out of 25 patients (68%) had an optimal lag screw positioning, classified as center–center [34]. However, a cadaveric study conducted by Hwang et al. in 2012 suggested that a center–inferior position provides a mechanical advantage over the center–center position under loading conditions [35]. In their 2014 study, Turgut et al. recommended either a center–center or center–inferior position for lag screws placed into the femoral neck [3]. In their 1992 publication, Parker et al. identified the posterior–superior quadrant as the least favorable location regarding the cut-out risk [36]. In our study, the center–center placement was predominant across all surgical techniques. The center–superior positioning was the second most common in Groups I and III, while the second most frequent positioning in Group II was posterior–superior.

In our study, although no statistically significant differences were observed between the groups according to the Baumgaertner and Ikuta classifications, Group 1 achieved better TAD values through the placement of longer lag screws and compression screws. In other words, while the fracture reduction quality was similar across the groups, higher-quality fixation was more frequently achieved in Group 1, where a traction table was used. In Abulsoud et al.’s study involving 96 patients, the lateral decubitus and traction table positions were compared, and no statistically significant differences were observed in terms of reduction or fixation [37]. However, one of the few studies in the literature comparing three different patient positions reported similar results to ours, with better outcomes observed in the traction table group [38].

Our study had limitations, including its retrospective design, possible selection bias, and limited generalizability due to regional practice patterns or equipment variability. Multicenter, prospective studies conducted with different implant designs will provide valuable contributions to the literature.

## 5. Conclusions

In conclusion, each of the three surgical techniques can be selected based on the surgeon’s experience and intraoperative factors (the fracture type, history of previous orthopedic surgery, and the material resources available during the procedure). Among the reduction criteria analyzed in our study, the traction table method demonstrated statistically significant superiority (*p* = 0.000) in terms of the TAD, achieving an optimal average distance of 25.31 mm compared with the other two positions. Additionally, the collodiaphyseal angle in the traction table group (mean: 130.72°) was significantly closer (*p* = 0.000) to the targeted angle range compared with the supine position. Considering the modified Baumgaertner classification, a good reduction rate of 59.2%, an Ikuta subtype N rate of 69.4%, and a Cleveland–Bosworth center–center placement rate of 31% were observed in the lateral decubitus position. Based on these findings, we recommend operating using a traction table as the primary choice and the lateral decubitus position as a secondary option when conditions allow, as it may provide better fixation quality and, thus, reduce the risk of complications such as cut-out and mechanical failure. We would like to emphasize that this recommendation is applicable only when the technical conditions are suitable for the operating surgeon and the surgical team.

## Figures and Tables

**Table 1:** Comparison of groups according to AO, modified Baumgartner, and Ikuta classifications.

		Group I	Group II	Group III	*p*
AO classification	31A1:	24 (33.8)	20 (40.8)	13 (35.1)	0.102
	31A2:	35 (49.3)	19 (38.8)	23 (62.2)	
	31A3:	12 (16.9)	11 (20.4)	1 (2.7)	
Modified Baumgaertner classification	Acceptable	24 (33.8)	18 (36.7)	10 (27)	0.420
	Good	45 (63.4)	29 (59.2)	23 (62.2)	
	Bad	2 (2.8)	2 (4.1)	4 (10.8)	
Ikuta classification	Subtype N	46 (64.8)	34 (69.4)	24 (64.9)	0.879
	Subtype P	12 (16.9)	9 (18.4)	8 (21.6)	
	Subtype A	13 (18.3)	6 (12.2)	5 (13.5)	

**Table 2:** Comparison of surgical technique with DAP, DLAT, TAD, and collodiaphyseal angle.

	Group I	Group II	Group III	*p*
D_AP_	13.88 ± 4.48	15.72 ± 5.68	15.20 ± 6.72	0.156
D_LAT_	11.43 ± 4.10	16.21 ± 7.08	17.11 ± 6.30	0.000
TAD	25.31 ± 6.62	31.91 ± 11.32	31.79 ± 11.14	0.000
Collodiaphyseal angle	130.72 ± 6.38	130.29 ± 8.04	124.19 ± 6.41	0.000

**Table 3:** Comparison of lag screw and compression screw lengths according to surgical technique.

	Group I	Group II	Group III	*p*
Lag screw length	97.21 ± 6.11	89.59 ± 7.06	93.78 ± 6.50	0.000
Compression screw length	92.18 ± 6.37	85 ± 6.9	88.78 ± 6.50	0.000

## Data Availability

The dataset generated and/or analyzed during the current study is available from the corresponding author.

## Abbreviations

The following abbreviations were used in this manuscript:

DAP	Anterior–posterior distance
DLAT	Lateral distance
PFN	Proximal femoral nail
TAD	Tip–apex distance
